# Long-term changes in hazardous heat and cold stress in humans: multi-city study in Poland

**DOI:** 10.1007/s00484-020-02069-7

**Published:** 2021-01-21

**Authors:** Magdalena Kuchcik, Krzysztof Błażejczyk, Agnieszka Halaś

**Affiliations:** 1 Climate Impacts Laboratory, Institute of Geography and Spatial Organization Polish Academy of Sciences, Twarda 51/55, 00-818 Warsaw, Poland; 2 Past Landscape Dynamic Laboratory, Institute of Geography and Spatial Organization Polish Academy of Sciences, Twarda 51/55, 00-818 Warsaw, Poland

**Keywords:** Hazardous heat and cold, UTCI, Regional changes, Poland, 68-year study

## Abstract

Significant changes in climate variables in the last decades resulted in changes of perceived climate conditions. However, there are only few studies discussing long-lasting changes in bioclimatic conditions. Thus, the purpose of this paper is to present the temporal and spatial distribution of hazardous heat and cold stress conditions in different regions of Poland. Its focus is on long-lasting changes in such conditions in the period 1951–2018. To assess changes in hazardous thermal stress conditions, the Universal Thermal Climate Index (UTCI) was used. UTCI values at 12 UTC hour (respectively 1 pm winter time, 2 pm summer time) were calculated daily based on air temperature, relative humidity, total cloud cover and wind speed at 24 stations representing the whole area of Poland. We found that the greatest changes were observed in minimum (1.33 °C/10 years) and average (0.52 °C/10 years) UTCI values as well as in cold stress frequency (− 4.00 days per 10 years). The changes vary seasonally and regionally. The greatest increase in UTCImin and decrease in cold stress days were noted from November to March and had the highest values in north-east and east Poland, and also in the foothills of the Carpathian Mountains. The trends in maximum UTCI are much smaller and not always positive. The spatially averaged trend in UTCImax for Poland as a whole was 0.35 °C/10 years and the increase in heat stress days was 0.80 days/10 years. The highest increases in UTCImax and heat stress days were noted in eastern and south-eastern Poland.

**Supplementary Information:**

The online version contains supplementary material available at 10.1007/s00484-020-02069-7.

## Introduction

Human beings are permanently under the influence of atmospheric stimuli which impact individual systems and organs in the body. Special attention is usually paid to the so-called “thermal environment”, which comprises both atmospheric heat exchange conditions (stress) and physiological response (strain) (Jendritzky et al. 2012). The magnitude of thermal impacts depends not only on air temperature but also on solar radiation, air humidity and wind speed, which together create man’s bio-thermal environment (Köppe et al. 2004; Gasparrini et al. 2015). Human organisms react to ambient stimuli and endeavour to balance the heat exchange and to preserve thermal equilibrium of the body core.

Balancing the human heat budget in variable atmospheric conditions is achieved by the autonomous thermoregulatory system, additionally supported by behavioural adaptation (Havenith 2001; Glossary 2003; Kenney and Munce 2003; Parsons 2003). In hot conditions, heat equilibrium of the body is mainly regulated through increased bodily sweating and consequent evaporation-induced cooling (Elizondo and Bullard 1971; Givoni and Goldman 1973; Kenney 1985; Hajat et al. 2007; Cheshire 2016). In a cold environment, heat balance is regulated by reduction of heat loss from the body (through vasoconstriction) and by production of heat by shivering (le Blanc 1975; Clark and Edholm 1985; Holmér 1988; Guyton and Hall 2006). Pappenberger et al. (2015) have presented global distribution of thermal stress. They also validated the influence of particular meteorological variables. They have found that UTCI is mostly dependent on changes in air temperature. Weaker influence is observed for downward solar and thermal radiation. The weakest relationships were found for wind speed (*v*) and air humidity (*f*). Bröde et al. 2013 reported that impacts of *v* and *f* changed significantly at different air temperatures.

Several biometeorological indicators of heat exposure, considering physical properties of the ambient environment, have been developed (Parsons 2003; Epstein and Moran 2006; Błażejczyk et al. 2012; de Freitas and Grigorieva 2017). In bioclimatic research, different indicators are applied to study various aspects of climate impact on humans, both individuals and society. In the last decade, the Universal Thermal Climate Index (UTCI), which defines thermal stress in humans, has been more and more frequently applied in bioclimatic research. UTCI is used, for instance, in research on mortality and morbidity (Nastos and Matzarakis 2012; Urban and Kyselý 2014; Burkart et al. 2016; Kuchcik 2017; Błażejczyk et al. 2018), work effectiveness (Bröde et al. 2013), assessment of bioclimatic potential for tourism and recreation (Błażejczyk and Kunert 2011), evaluation of urban bioclimate (Bröde et al. 2013; Błażejczyk et al. 2014) and many others.

In the last decades, general climate research has concentrated on identifying climate changes and their causes (IPCC 2018). Indisputably, an increase in air temperature may be observed all over the world. The increasing trend has accelerated in the last 30 years (Gajic-Capka and Zaninovic 1997; Piotrowicz 2007; Wibig et al. 2009a, 2009b; Kuchcik 2017).

Changes in air temperature and other climate variables lead to changes in bioclimatic conditions. As bioclimatic indicators require the input of a wide range of meteorological data and their preparation and calculation are time consuming, they are not frequently analysed. Up to now, the longest series of changes in bioclimatic variables were developed for Cracow (Poland) by Błażejczyk and Twardosz (2010). The authors analysed several indices (WCT, PST, Humidex, PhS) for the period 1826–2006. Analyses covering shorter periods have been prepared for Vienna (Koch et al. 1992), China (Kong et al. 2019), Serbia (Pecelj et al. 2017; Stojićević et al. 2016), the Adriatic coast (Zaninovic and Matzarakis 2007), Hungary (Németh 2011), and Croatia and Slovenia (Zaninovic et al. 2006), as well as for different regions of Poland (Owczarek 2009; Kuchcik et al. 2013). So far, only few studies present long-term historical changes in UTCI (Okoniewska and Więcław 2013; Cheung and Hart 2014; Błażejczyk et al. 2015, 2018; Kuchcik 2017; Owczarek 2019; Tomczyk and Owczarek 2020).

Therefore, the aim of the present paper is to analyse the temporal and spatial distribution of hazardous heat and cold stress conditions in Poland. Special attention is paid to their long-term changes, both annual and monthly, in the period 1951–2018 in different regions of Poland.

## Methods

**Fig. 1:**
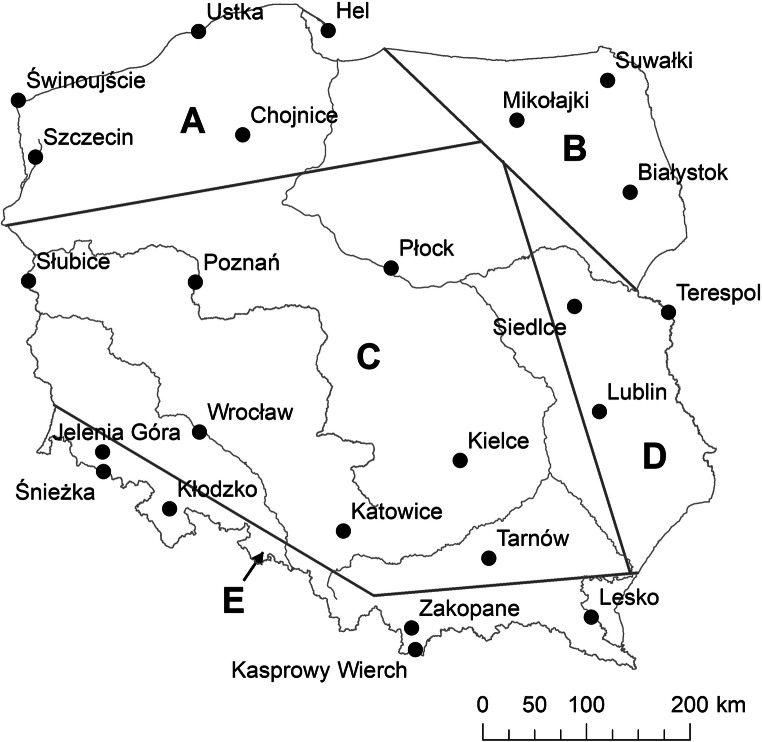
Stations providing data used in study; symbols explanation in the text. Source: own elaboration

To assess changes in hazardous thermal stress conditions in Poland, the Universal Thermal Climate Index (UTCI; Błażejczyk et al. 2012; Bröde et al. 2012) was used. UTCI reflects the intensity of heat and cold stress in humans, which are physiological reactions of an organism to atmospheric stimuli. For this purpose, daily values of UTCI at 12 UTC (respectively 1 pm winter time, 2 pm summer time) were calculated based on air temperature, relative humidity, total cloud cover and wind speed. Time in the middle of the day corresponds to the highest activity of people in Poland. The analysed period covers 68 years, from 1951 to 2018; data were collected at 24 stations selected to represent the whole area of Poland (Fig. 1). UTCI was calculated using the BioKlima 2.6 software package.

We examined minimum (UTCImin), mean (UTCIavg) and maximum (UTCImax) values and frequencies of selected thermal stress categories. In the analysis, all stress categories in the range UTCI ≤ − 13.0 °C (strong, very strong and extreme cold stress) were grouped as cold stress (CS), while UTCI > 32 °C (strong, very strong and extreme heat stress) was defined as heat stress (HS). The thresholds were chosen according to previous mortality studies in Poland in which only those conditions were burdensome for the body and aggravated mortality. Under the other stress categories: “no thermal stress”, “slight cold stress”, “moderate heat/cold stress”, mortality in Poland was on the average level or decreased (Błażejczyk et al. 2015, 2018; Kuchcik 2017, 2020).

To compare changes in UTCI characteristics, we used 10-year trends. The relative trends in the increase/decrease in the percentage of hot and cold days were calculated in relation to 1951. The trends and their statistical significance were verified with Stragraphics Centurion XVI, version 16.2.04.

In the paper, only trends that are statistically significant at *p* ≤ 0.05 are taken into account. All trend values for individual stations are presented in supplementary materials. We also calculated spatial averages of monthly and annual trends for Poland as a whole and for specific areas (as the averages from all stations in given region). In the averaging procedure, only the trends that were statistically significant at *p* ≤ 0.05 were taken into account. The meteorological stations used in this research can be divided into five groups according to the specific patterns of their geographical environment: northern (A, including coastal, influenced by the Baltic Sea), north-eastern (B, exposed to advection of arctic and continental air masses), central (C, mainly lowlands, which are strongly influenced by traversing of oceanic and continental air masses), eastern (D, with increased advection of polar continental air) and southern (E, mountainous, where vertical changes of meteorological parameters create the climate and bioclimate of the area).

## Spatial distribution of thermal stress in Poland

**Fig. 2:**
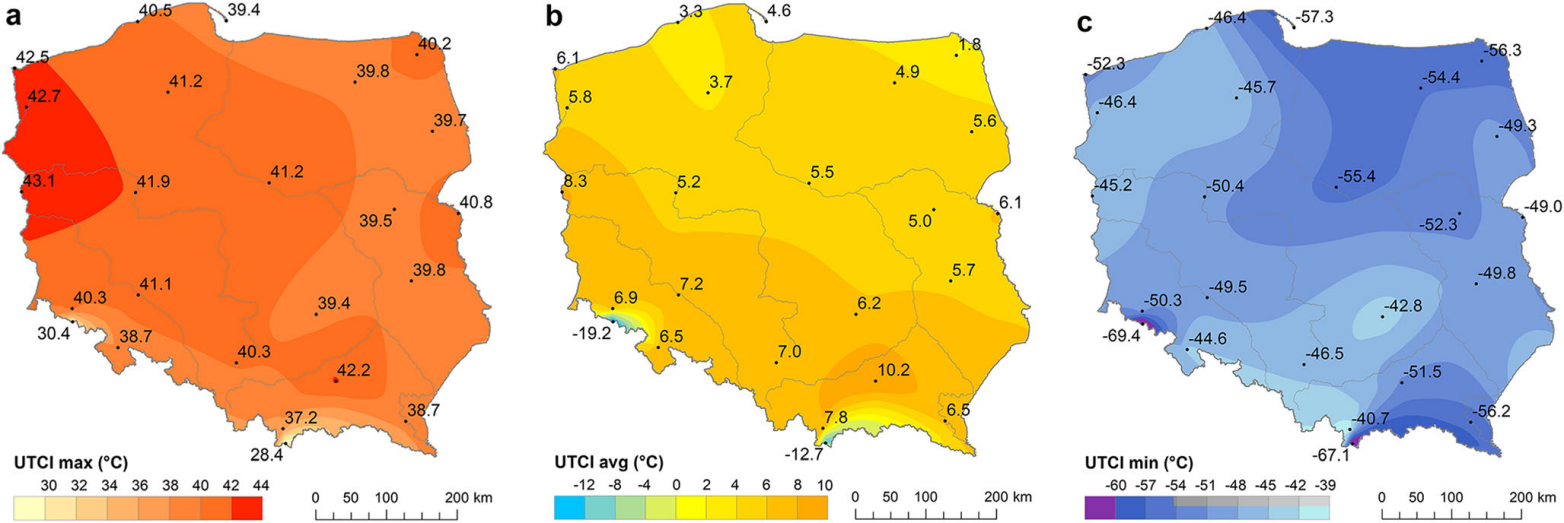
The highest (**a**), yearly average (**b**) and the lowest (**c**) values of UTCI, 1951–2018. Source: own elaboration

Thermal stress conditions in Poland are spatially differentiated. Individual UTCI characteristics (maximum, minimum and mean values and the number of days with heat and cold stress conditions) changed regionally. The highest UTCIavg values (10.2 °C) were noted in the south in the Sandomierski Basin (Tarnów). The spatial distribution of yearly UTCIavg follows diagonal belts running from the north west to the south east: the lowest values were recorded in the north east and the highest ones, in the south and south west, except in mountainous areas (Fig. 2). The highest values (> 40 °C) for UTCImax were noted in western and central Poland and the lowest ones, in mountain regions, especially on summits. The opposite distribution occurred for UTCImin. In this case, the lowest values (< − 55 °C) were observed in north-eastern and mountain regions (especially the Carpathians). Relatively high UTCImin values were noted in north-western Poland and in the central Upland Region (Kielce, Katowice). One of the reasons for such a distribution is that north-eastern and eastern Poland is influenced by frequent advections of arctic and polar continental air masses, but in western and north-western areas, increased frequency of oceanic air masses is visible (Fig. 2).

**Fig. 3:**
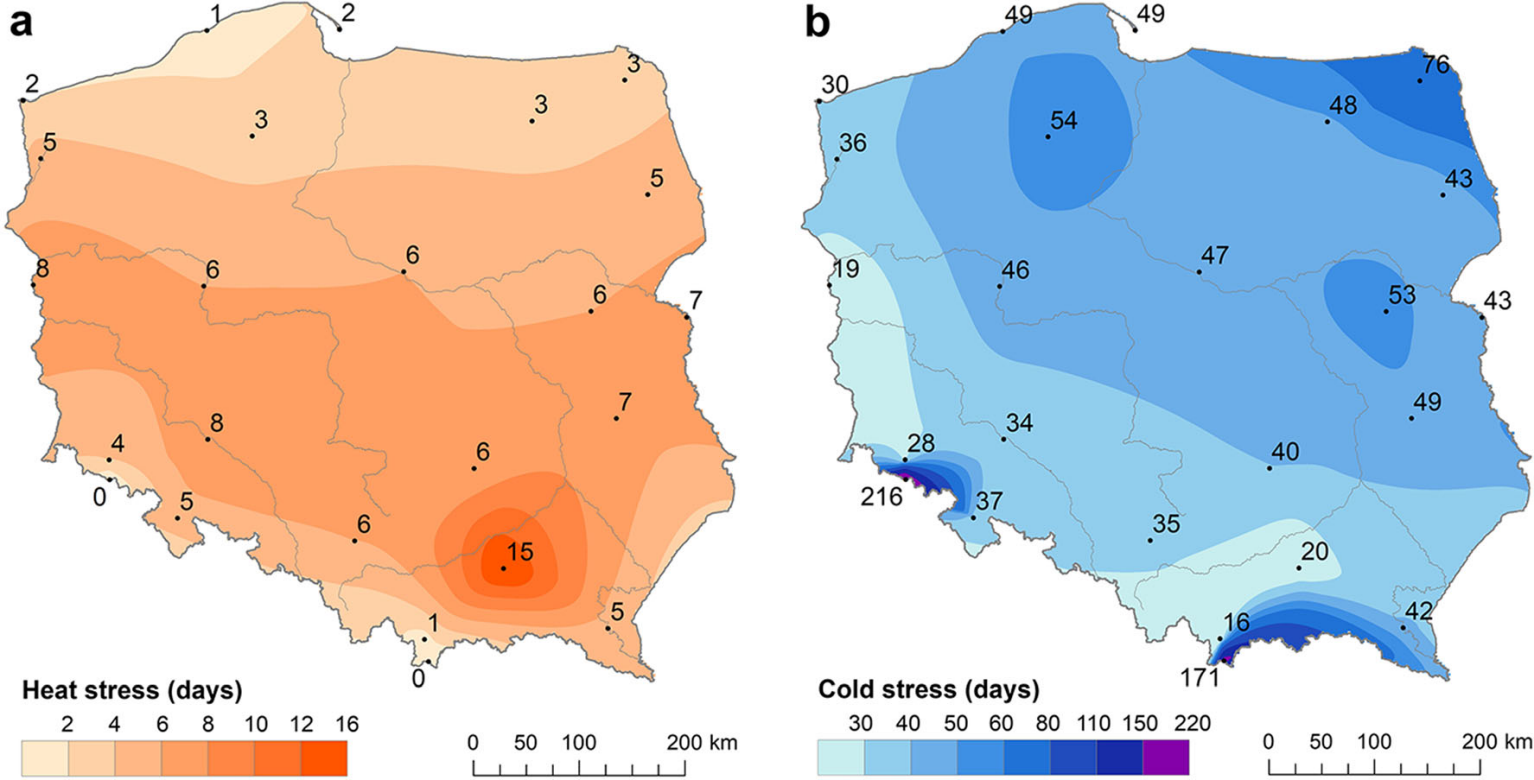
Average number of heat stress (**a**) and cold stress (**b**) days, 1951–2018. Source: own elaboration

On average, 12–21% of days in the year (i.e. 43–76 days) represented cold stress and only 1–2% (3–5 days) showed heat stress. This is the most visible in the mountain stations where very low values of UTCI were registered. Thus, these regions (especially mountain summits) are characterised by greater frequency of cold stress days. Heat stress days are almost never observed there. High UCTImax values on the Baltic coast are not reflected in the frequency of heat stress days. Coastal locations are characterised by lower frequency of heat stress (0.4–0.6% of the year, i.e. 1–2 days). In lowland stations, thermal stress varies significantly. In western locations, the impact of oceanic air masses results in the small frequency of cold stress and heat stress days (19 and 8 days per year, respectively). The most southerly parts of the lowland area (Tarnów) can be considered the warmest in Poland, with a significantly high frequency of heat stress (on average 15 days yearly, but 35 days in 2012 and 2015) (Fig. 3).

## Changes in UTCI values

**Fig. 4:**
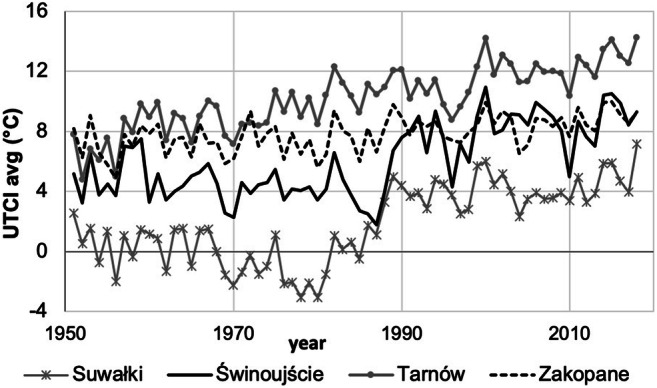
Multiannual changes in mean yearly values of UTCI in selected stations in Poland, 1951–2018. Source: own elaboration

Over the studied period (1951–2018), UTCI values have changed. A general overview of UTCIavg shows its increasing trends. The intensity of changes accelerated in the second half of the studied period. Depending on station, this acceleration began between 1980 and 1995. The exception is mountain stations (e.g. Zakopane), where a slight gradual increase in UTCIavg was observed over the whole studied period (Fig. 4).

**Fig. 5:**
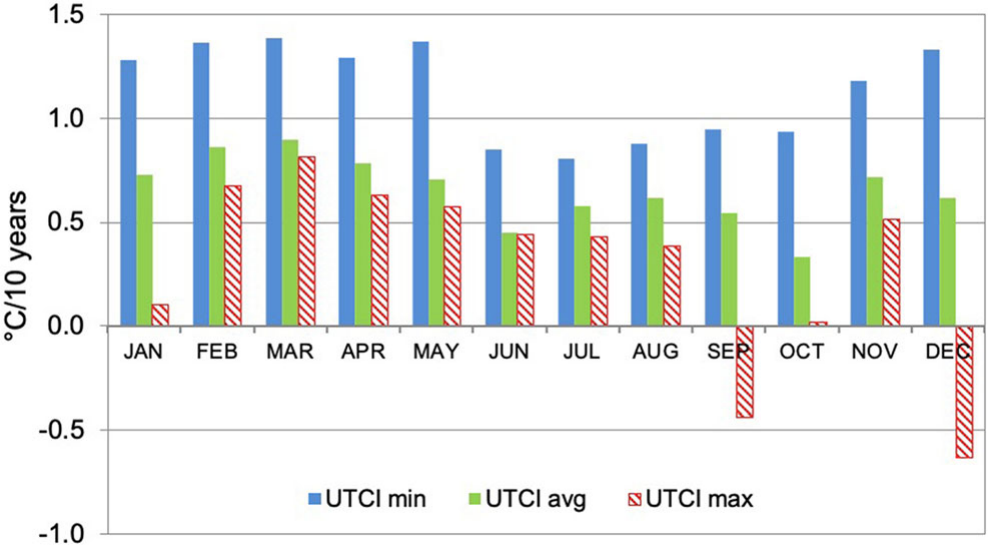
Spatially averaged (for all stations of the whole area of Poland) monthly trends in minimum (UTCImin), average (UTCIavg) and maximum (UTCImax) UTCI values, 1951–2018. Source: own elaboration

The changes in thermal stress patterns in Poland differ depending on the analysed characteristic. In terms of individual months, spatially averaged statistically significant trends for the whole of Poland were the highest for UTCImin and they fluctuated from 0.81 °C/10 years in July to 1.39 °C/10 years in March. Lower trends were observed for UTCIavg, varying from 0.33 °C/10 years in October to 0.90 °C/10 years in March. The trends were the smallest for UTCImax and did not exceed 0.82 °C/10 years (in March). In the case of two months (September and December), UTCImax trends were even negative. In general, the highest trends occurred from February to May (Fig. 5).

The trends in UTCImin were noticeable, and statistically significant (*p* ≤ 0.05) changes for annual UTCImin values were observed at 79% of stations. The spatially averaged annual trend for the whole of Poland was 1.33 °C/10 years but varied from 0.55 °C/10 years in Kielce to 2.19 °C/10 years in Tarnów. Significant monthly changes in UTCImin were observed in the majority of studied stations (52–76%, depending on the month) and they usually occurred in more than 6 months of the year. Several exceptions were observed: stations like Ustka or Lublin where significant changes were found only for one month, or the month of October when only 24% of stations recorded significant changes (Table B in supplementary materials).

UTCImin trends vary regionally. Their highest values were found for north-eastern Poland and their lowest ones in the northern coastal region. Stations in central Poland varied the most in terms of UTCImin trends. In this group of stations, both the lowest (0.46 °C/10 years, June in Poznań) and highest (2.65 °C/10 years, March in Tarnów) monthly trends were observed. Seasonal changes in UTCImin trends were the most visible in the north-eastern area where they varied from 0.74–0.75 °C/10 years in June and July to 1.85 °C/10 years in February (Table 1).

**Table 1:** Statistically significant 10-year trends in monthly and yearly minimum values of UTCI (spatially averaged for individual areas), 1951–2018

Region	Jan	Feb	Mar	Apr	May	Jun	Jul	Aug	Sep	Oct	Nov	Dec	Year
Northern	0.99	1.40	1.22	1.15	1.41	0.81	0.79	0.80	0.95	0.93	0.92	1.25	1.15
North-eastern	1.35	1.85	1.65	1.51	1.38	0.75	0.74	0.93	0.96	1.07	1.67	1.65	1.75
Central	1.42	1.33	1.58	1.54	1.44	0.80	0.90	0.82	0.97	0.90	1.21	1.23	1.26
Eastern	0.98	1.33	1.36	1.35	1.40	0.96	0.64	0.81	0.94		1.02	1.26	1.20
Southern (Mts.)	1.43	1.06	1.08	0.85	1.23	0.92	0.77	0.99	0.92	0.75	1.17	1.26	1.37

An empty cell indicates that there was not any station in the given area with trend value statistically significant at *p* ≤ 0.05

Source: own elaboration

The spatially averaged UTCIavg annual trend for Poland as a whole is 0.52 °C/10 years. The trends were significant at 71% of stations; however, they differed between stations, regions and seasons. The highest annual trends were found for Suwałki (0.90 °C/10 years), Tarnów (0.89 °C/10 years) and Świnoujście (0.80 °C/10 years). Their lowest values (< 0.3 °C/10 years) were recorded for Chojnice, Śnieżka, Kasprowy Wierch and Słubice (Table A in supplementary materials). In a yearly cycle, the highest monthly trends (> 0.7 °C/10 years) were noted from January to May and in November. October was the month with the lowest UTCIavg trend (0.33 °C per 10 years) (Fig. 5). The trends were significant during at least 9 months only in 1/3 of stations, and in 7 stations, the trends were significant during 1–3 months. In Ustka, no month with a significant UTCIavg trend was observed. In the majority of mountain stations, the period from April to August was characterised by significant monthly trends.

With respect to regional variability of UTCIavg trends, the highest mean annual value (0.73 °C/10 years) occurred in north-eastern Poland and the lowest one (0.34 °C/10 years) at mountain stations. Like the trends in individual stations, regional trends differed seasonally as well. In the majority of areas, their lowest values occurred in June and October. In mountain areas, October was a month with a decreasing UTCIavg trend (Table 2).

**Table 2:** Statistically significant 10-year trends (°C/10 years) for monthly and yearly mean values of UTCI (spatially averaged for individual regions), 1951–2018

Region	Jan	Feb	Mar	Apr	May	Jun	Jul	Aug	Sep	Oct	Nov	Dec	Year
Northern	0.72	0.77	0.80	0.82	0.77	0.44	0.56	0.52	0.50	0.54	0.68	0.73	0.52
North-eastern	0.72	0.92	0.95	1.03	0.94	0.39	0.70	0.75	0.58	0.52	0.86	0.73	0.73
Central	0.89	1.00	0.98	0.87	0.70	0.53	0.60	0.61	0.51	0.43	0.74	0.61	0.57
Eastern	0.55	0.70	0.80	0.80	0.63		0.49	0.59			0.67	0.42	0.53
Southern (Mts.)	0.66			0.52	0.54	0.41	0.52	0.63		− 0.35	0.42	0.60	0.34

An empty cell indicates that there was not any station in the given region with trend value statistically significant at *p* ≤ 0.05

Source: own elaboration

Maximum UTCI values changed significantly in only 38% of the studied stations (with respect to a yearly trend). In individual months**,** the number of such stations is even smaller and varies from 1 station (4%) in February, March and November to 8 stations (32%) in August. For 9 of the stations covered, the spatially averaged yearly UTCImax was only 0.35 °C/10 years and varied from 0.22 °C/10 years in Siedlce to 0.53 °C/10 years in Suwałki. Among mountain stations, a yearly increasing trend in UTCImax was statistically significant only in Zakopane. In the period from September to March**,** the monthly trends were positive and significant only at a few stations, but most of them indicated decreasing UTCI maximums (Table C in Supplementary materials).

Because of the small number of stations with statistically significant changes in UTCImax, no regional differences in their annual run could be found. August was the only month with significant values of positive trends in UTCImax in all regions of Poland. For that month, the trend values vary from 0.34 °C/10 years in the mountains to 0.43 °C/10 years in the north (Table 3).

**Table 3:** Statistically significant 10-year trends (°C/10 years) for monthly and yearly maximum UTCI values (spatially averaged for individual regions), 1951–2018

Region	Jan	Feb	Mar	Apr	May	Jun	Jul	Aug	Sep	Oct	Nov	Dec	Year
Northern	0.78		0.82	0.63	0.67		0.50	0.43	− 0.41		0.51	− 0.53	0.39
North-eastern	0.51			0.92	0.57	0.46	0.56	0.38		0.54			0.38
Central	− 0.58	0.67		0.49	0.54	0.35	0.38	0.38	− 0.46	− 0.49		− 0.58	0.34
Eastern								0.39					0.26
Southern (Mts.)	− 0.70				0.50	0.50	0.32	0.34				− 0.78	0.35

An empty cell indicates that there was not any station in the given region with trend value statistically significant at *p* ≤ 0.05

Source: own elaboration

## Changes in hazardous thermal stress conditions

For humans, physiological reactions to heat and cold stress are more important than UTCI values themselves: this is why changes in the frequency (number of days per year) of selected UTCI categories were studied.

**Fig. 6:**
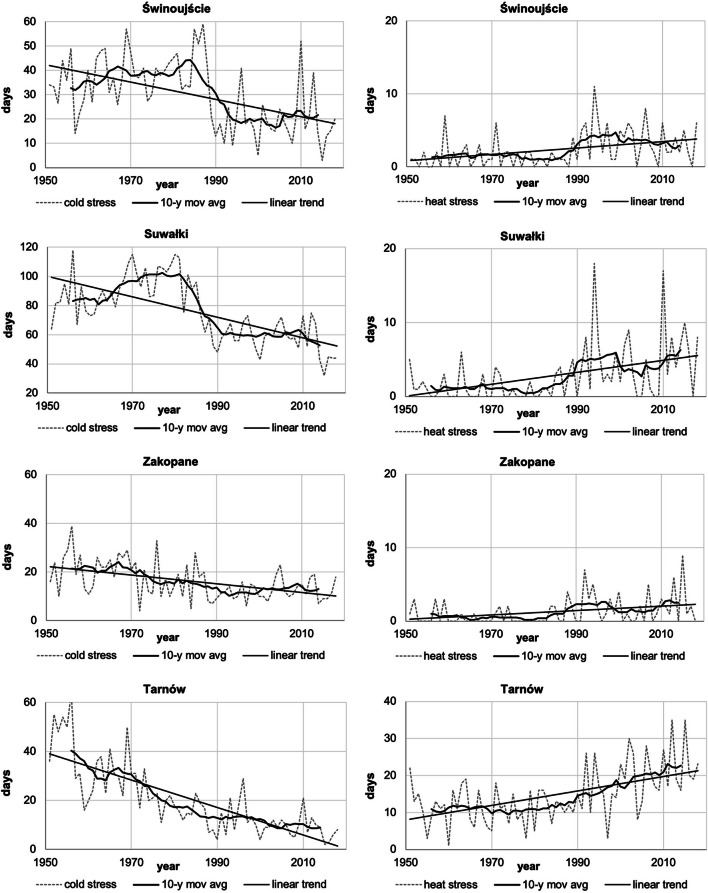
Changes in heat and cold stress days in selected cities representing different regions (with 10-year moving average and trend), 1951–2018. Source: own elaboration

In the analysis of year-to-year changes in UTCI stress categories, it should be emphasised that the fluctuations of cold stress days (CS) were 3 times bigger than those of heat stress days (HS), especially at stations located in northern and eastern Poland. This is illustrated with the example of four cities that represent the northern coastal region (Świnoujście), the north-east region (Suwałki), the southern mountainous region (Zakopane) and the central region (Tarnów—situated in the very south of this region) (Fig. 6).

The rise in UTCI values results in the increase of HS (most spectacularly in Tarnów and Suwałki). The lowest changes in the number of HS were observed on the sea coast. There were periods of very high HS frequency (e.g. the years 1990–2000) and periods of lower frequency (e.g. the 1970s and 1980s). Even in the most recent years, the coast and the north east recorded summers without HS, like in 2009 or 2017.

Within the designated regions, the maximum yearly number of HS varied significantly. In the north-east region, it varied from 12 to 18 in Suwałki (in 1994); in the northern region, it varied from 5 in Ustka, the station situated almost on the beach, to 23 in Szczecin. In the central area, the maximum number of HS was also highly varied: from 16 in Płock to 35 in Tarnów (in 2015). The mountainous region was also diversified with respect to this category, with 9 HS recorded in Zakopane and 24 in Lesko. As expected, on the summit stations (Śnieżka and Kasprowy Wierch), heat stress did not occur. Only at three stations representing the eastern region were the maximum numbers of HS similar to each other (20–21 days), but they occurred in different years (1951, 1994, 2015).

The highest changes in HS were noted on the western, northern and north-eastern edges of Poland (e.g. Świnoujście, Suwałki), the areas frequently reached by fresh air masses i.e. polar maritime air, and arctic and polar continental air.

The decrease in CS is high and constant (as in Tarnów) or clear but highly variable, as in Suwałki, which was characterised by periods of high frequency of CS (1970–1984; even 115 days in 1980), followed by its low occurrence (48 days in 1990 and only 32 in 2015) (Fig. 6).

**Fig. 7:**
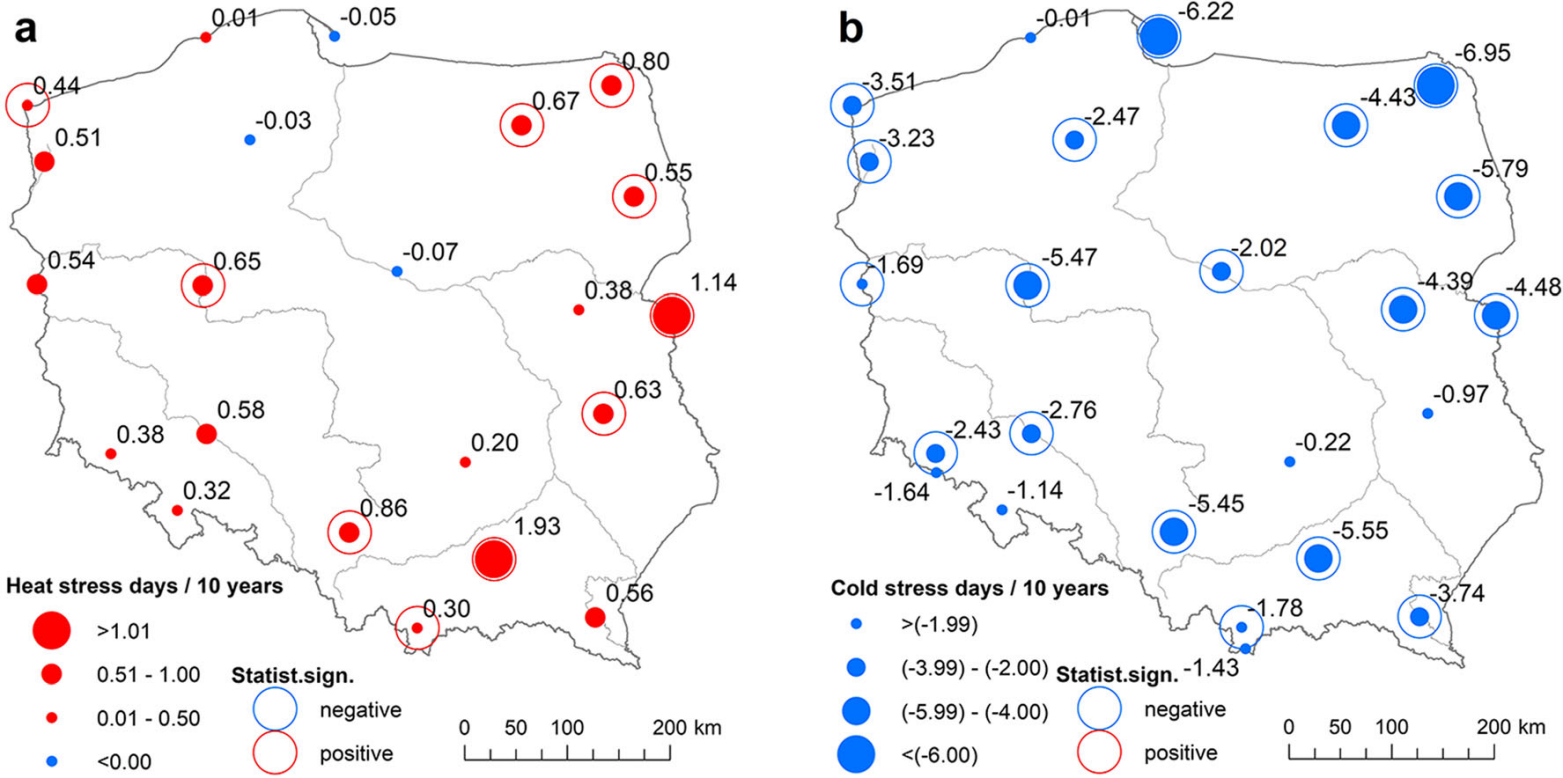
10-year trends in heat (**a**) and cold (**b**) stress days, 1951–2018. Statistical significance at *p* ≤ 0.05. Source: own elaboration

The trends in heat and cold stress days at individual stations are presented in Fig. 7. Above all, at all stations, a negative trend in the annual number of CS was observed. The greatest reduction in CS was observed in Suwałki (− 6.9 days/10 years), Hel (− 6.2 days/10 years) and Białystok (− 5.8 days/10 years). Trends in the mountainous area were much smaller and statistically insignificant. At 18 of the 24 analysed stations, the decrease in the number of CS is statistically significant (Fig. 7b).

The increase in HS is much lower than the reduction in CS. In the south, it reached 1.9 days/10 years in Tarnów and 0.6–1.1 days/10 years in the east and north east of Poland (Terespol, Suwałki, Białystok, Lublin). Although the absolute values of the trends may not seem spectacular, the trend of 0.8 heat stress days/10 years noted in Suwałki means that the frequency of such days rose from 1.4 in the years 1951–1960 to 6.3 in the last 10 years (Fig. 6). The lowest changes in the number of HS were observed on the sea coast. However, most stations were characterised by small, statistically insignificant increases in HS—significant increases were noted at 10 of the 24 analysed stations. At a few stations located on the coast and in the very centre of Poland, in the Vistula river valley, no significant trends were recorded, and at three stations, they were even negative, which means a small decrease in HS (Fig. 7a).

Moving towards a generalisation of the results, spatially averaged trends within the regions show the highest annual trend in HS in the central region (1.15 days/10 years) and the lowest in the north and in the mountains, which only confirms previous results. August is the only month in which all regions were characterised by positive and significant trends, reaching 0.55 heat stress days/10 years in the central region (Table 4).

**Table 4:** Statistically significant 10-year trends in heat stress days (days/10 years) spatially averaged for individual regions, 1951–2018

Region	May	Jun	Jul	Aug	Sep	Year
Northern	− 0.02		0.22	0.22		0.44
North-eastern		0.12	0.39	0.31		0.73
Central	0.13	0.28	0.52	0.55		1.15
Eastern			0.39	0.51	0.06	0.88
Southern (Mts.)		0.07		0.38	− 0.08	0.46

An empty cell indicates that there was not any station in the given region with trend value statistically significant at *p* ≤ 0.05

Source: own elaboration

All trends presented in Fig. 7b indicate that the highest average decreasing trend in CS would characterise the north-eastern region. Spatially averaged yearly trends in CS reached − 5.71 days/10 years in the north east and only − 2.57 days/ 10 years in the south. Significant decreasing trends in CS occur in all regions from November to March. In the mountainous region, they also occur in summer, reaching, in August, − 0.75 days/10 years at Śnieżka and − 0.29 days/10 years at Kasprowy Wierch (Table 5, Table D in Supplementary materials).

**Table 5:** Statistically significant 10-year trends in cold stress days (days/10 years) spatially averaged for individual regions, 1951–2018

Region	Jan	Feb	Mar	Apr	May	Aug	Sep	Oct	Nov	Dec	Year
Northern	− 1.11	− 0.94	− 0.73	− 0.40	− 0.08				− 0.58	− 0.90	− 3.86
North-eastern	− 0.97	− 1.07	− 1.05	− 0.35	− 0.09			− 0.21	− 1.05	− 0.99	− 5.71
Central	− 1.07	− 0.96	− 0.81	− 0.20	− 0.02			− 0.06	− 0.54	− 0.93	− 3.83
Eastern	− 0.89	− 0.94	− 0.81	− 0.25					− 0.63	− 0.88	− 4.42
Southern (Mts.)	− 0.58	− 0.57	− 0.48		− 0.51	− 0.52	− 0.02		− 0.47	− 0.70	− 2.57

An empty cell indicates that there was not any station in the given region with trend value statistically significant at *p* ≤ 0.05

Source: own elaboration

**Fig. 8:**
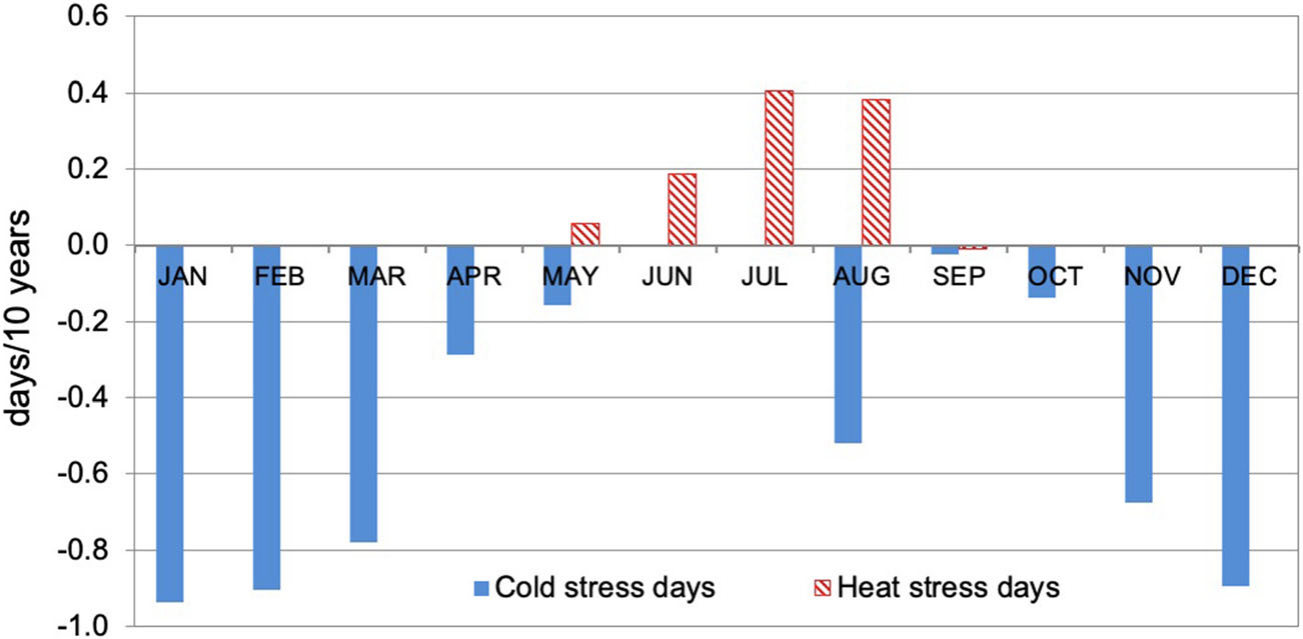
Spatially averaged (for all stations analysed) monthly trends in cold and heat stress days 1951–2018. Source: own elaboration

Spatially averaged statistically significant at *p* ≤ 0.05 trends for Poland as a whole confirm the previous pattern, and also give a very general picture that could be useful for future comparisons between countries. The average trend in CS is − 4.0 days/10 years and it fluctuates from − 0.02 days/10 years in September to − 0.94 days/10 years in January. On average, in the whole area of Poland, the highest decreasing trends occur in the coldest months (December–February). In the summer, cold stress days occur only in the mountains, at the summit stations, and there is a clear decreasing trend, especially in the Sudetes (Fig. 8).

The spatially averaged statistically significant trend for HS in Poland as a whole is 0.80 days/10 years, and it varies from 0.06 days/10 years in May to 0.41 days/10 years in July (Fig. 8).

## General assessment of changes in bioclimatic conditions

The analysis of the changes in bioclimatic conditions shows significant increases mainly in minimum and mean values of UTCI in the period studied. UTCImin showed significant increases in the whole area of Poland, including the mountains, but especially in the north east, where UTCImin growth is on average 1.75 °C/10 years. The highest increase was observed in Tarnów, situated in the south east of the central region, with a positive trend of 2.19 °C/10 years. Positive trends, varying from 0.64 to 1.85 °C/10 years depending on month and region, occur in every region and in all months, except for October in the eastern region.

A rise in maximum UTCI values is not so clear or common in Poland. Significant trends are the highest in the northern and north-eastern regions and their yearly values are 0.38–0.39 °C/10 years. The highest trend in UTCImax reached 0.92 °C/10 years in Suwałki in April and the yearly average trend there was 0.53 °C/10 years. Only in August significant trends occurred in all regions. In September and December, the spatially averaged trends in UTCImax for Poland as a whole were negative.

The spectacular increase in UTCImin resulted foremost in a significant decrease in cold stress days. The spatially averaged trend for Poland was − 4.0 days/10 years, but in north-eastern Poland, the trend was − 5.71 days/10 years. In general, at all analysed stations, a negative trend in cold stress days was observed, but was statistically significant at *p* ≤ 0.05 in 75% of them, varying from − 1.69 days/10 years in Słubice on the west Polish border to − 6.95 days/10 years in Suwałki in the very north east of the country.

The increasing trends in heat stress days were much smaller than the decreasing trends in cold stress, and significant at only 42% of stations. The increase in heat stress days was noted mainly in July and August; however, only in August did it occur in all regions. The spatially averaged trend for Poland as a whole was 0.80 days/10 years. Its highest values were observed in central Poland (1.15 days/10 years), mainly due to the Tarnów station where the trend was 1.93 days/10 years.

On a regional level, the greatest increase in mean and minimum UTCI values and decrease in the frequency of cold days were observed in north-eastern and eastern Poland, but and in the foothills of the Carpathian Mountains. Tarnów has to be added to these regions because, after a huge rise in both minimum and maximum values of UTCI, it has become the warmest city among those analysed in terms of UTCI. In the central region, the changes were much smaller. The highest increases in UTCImax and heat stress days were noted in eastern and south-eastern Poland.

While UTCI is based on several meteorological parameters, the comparison of their trends in selected cities representing different regions was made. In the years 1951–2018 at all stations, significant rise of air temperature was noted from 0.26 °C/10 years in Zakopane (Mts. region) to 0.38 °C/10 years in Tarnów (the warmest area). In the entire period, significant decrease of wind speed occurred and amounted from − 0.07 m s^−1^/10 years in Zakopane to − 0.20 m s^−1^/10 years in Tarnów. At 3 stations, there is small but significant decrease of cloudiness. The exception is Zakopane where small increase was noted. The decrease of relative humidity is significant only in Zakopane and Tarnów where it reached − 0.88%/10 years (Table 6). The changes of parameters resulted in much higher rising trends of UTCImean value.

**Table 6:** 10-year annual trends of UTCIavg and their meteorological compounders (air temperature, wind speed, cloudiness, relative humidity) in selected cities representing different regions, 1951–2018

Parameter	Suwałki	Świnoujście	Tarnów	Zakopane
Air temperature (°C/10 years)	*0.32*	*0.30*	*0.38*	*0.26*
Wind speed (m s^−1^/10 years)	*− 0.18*	*− 0.11*	*− 0.20*	*− 0.07*
Cloudiness (octans/10 years)	*− 0.11*	*− 0.11*	*− 0.05*	*0.06*
Relative humidity (%/10 years)	− 0.03	− 0.20	*− 0.88*	*− 0.23*
UTCIavg (°C/10 years)	*0.90*	*0.80*	*0.89*	*0.30*

Italic values indicate trend statistically significant at *p* ≤ 0.05

## Discussion and conclusions

Previous research dealing with long-term changes in Poland’s bioclimate used various indicators, but UTCI was applied only in few papers. Błażejczyk and Twardosz (2002, 2010) analysed changes in bioclimatic conditions in Krakow in the nineteenth and twentieth centuries using physiological subjective temperature (PST). In the years 1826–2006, PST grew significantly in January by 0.95 °C per 100 years (Błażejczyk et al. 2012; the study shows a high correlation between PST and UTCI)

Since its final development in 2009, the Universal Thermal Climate Index has gained worldwide recognition and has been used in numerous climate-human studies, for which it was mainly created (e.g. Kuchcik et al. 2013; Morabito et al. 2014; Nastos and Matzarakis 2012; Urban and Kyselý 2014; Błażejczyk et al. 2015, 2018; Gao et al. 2018), and also in bioclimate change studies, e.g., in Hungary in the years 1971–2000 (Németh 2011), China in the years 1981–2010 (Chi et al. 2018), Europe as a whole (di Napoli et al. 2018) and across the world, including Poland. Some papers do not analyse the trends, observing only the values or the frequency of heat stress days, like in Lublin in the years 1952–2010 (Dobek and Krzyżewska 2015) or in Warsaw in the years 1998–2015 (Rozbicka and Rozbicki 2018).

Only a few studies have analysed the trends and the changes in UTCI values. An analysis of UTCI trends in the years 1975–2014 in the largest Polish cities confirms the results obtained in this paper: the highest and statistically significant fall in the number of cold stress days (reaching 3 days/10 years from 1975 to 1989) and rise in heat stress days were noted in north-eastern Poland, with only a significant fall in cold stress days on the coast (in Gdańsk, close to the Hel peninsula) and no rise in heat stress days, etc. (Kuchcik 2017). Another long-term analysis of heat stress days according to UTCI (1966–2015) in the whole area of Poland showed usually higher positive trends (Tomczyk and Owczarek 2019) e.g. an increase in heat stress days by 1.3 days/10 years in north-eastern Poland (Suwałki) versus 0.8 days/10 years in the years 1951–2018 or 1.4 days/10 years in Poznań over the years 1966–2015 versus 0.65 days/10 years in the present research. This is mostly because the shorter period 1966–2015 does not include cold mid 1950s and early 1960s which are in contrast included the period 1951–2018. Furthermore that work does not analyse cold stress days.

Błażejczyk et al. (2015) have analysed the average number of heat and cold stress days in Poland in the years 1966–2012, on the basis of data from a similar set of stations, but no regional spatial averages or monthly trends were calculated. However, the course of the yearly number of cold and heat stress days was obviously similar to that obtained in this work.

The results of this paper clearly indicate that the general increase in thermal stress in Poland depends mostly on changes in climate variables (temperature, humidity, cloudiness and wind speed) in the cold months. This is confirmed by trends in the frequency of thermal stress categories related to cold environments. The paper shows also that the changes of the UTCI value are much bigger that the changes of the meteorological parameters analysed separately which once again confirms the legitimacy of using biometeorological indicators in analyses rather than individual meteorological elements.

It should also be noted that changes in thermal stress conditions in Poland vary seasonally and regionally. This may be caused by seasonal patterns of air circulation and regional climate features of individual stations. More detailed studies on this topic are needed in the future.

## Supplementary information

ESM 1 (DOCX 52 kb)
