# Supplementary material for: Long-term changes in hazardous heat and cold stress in humans: multi-city study in Poland
Source: Int J Biometeorol. 2021 Jan 21;65(9):1567–78. doi: 10.1007/s00484-020-02069-7 (PMC8370959; doi:10.1007/s00484-020-02069-7)
Supplement: Supplementary file 1 — (DOCX 52 kb) [file 484_2020_2069_MOESM1_ESM.docx]

Table A. The values of trends of mean UTCI per 10 years, 1951-2018

|  | JAN | FEB | MAR | APR | MAY | JUN | JUL | AUG | SEP | OCT | NOV | DEC | YEAR |
| --- | --- | --- | --- | --- | --- | --- | --- | --- | --- | --- | --- | --- | --- |
| **North** | | | | | | | | | | | | | |
| Chojnice | 0.11 | 0.21 | 0.41 | **0.68** | **0.66** | 0.03 | 0.41 | **0.39** | 0.23 | -0.03 | 0.16 | 0.05 | **0.28** |
| Hel | **0.70** | **0.76** | **0.73** | **0.81** | **0.82** | 0.12 | **0.38** | **0.39** | 0.33 | 0.23 | **0.70** | **0.70** | **0.55** |
| Szczecin | **0.48** | **0.57** | **0.66** | **0.75** | **0.64** | 0.06 | **0.56** | **0.54** | 0.31 | 0.27 | **0.46** | 0.29 | **0.47** |
| Świnoujście | **0.98** | **0.98** | **1.01** | **1.03** | **0.95** | **0.44** | **0.74** | **0.78** | **0.50** | **0.54** | **0.89** | **0.76** | **0.80** |
| Ustka | -0.01 | 0.13 | -0.21 | -0.01 | 0.31 | -0.13 | 0.28 | 0.15 | 0.06 | -0.25 | 0.19 | -0.07 | 0.04 |
| **NORTH-EAST** | | | | | | | | | | | | | |
| Białystok | **0.78** | **0.93** | **0.96** | **0.83** | **0.86** | 0.14 | **0.62** | **0.62** | **0.47** | **0.44** | **0.96** | **0.66** | **0.69** |
| Mikołajki | **0.49** | **0.78** | **0.76** | **1.00** | **0.81** | 0.20 | **0.64** | **0.74** | **0.48** | 0.22 | **0.57** | 0.39 | **0.59** |
| Suwałki | **0.91** | **1.05** | **1.13** | **1.26** | **1.15** | **0.39** | **0.86** | **0.87** | **0.79** | **0.59** | **1.04** | **0.79** | **0.90** |
| **CENTER** | | | | | | | | | | | | | |
| Katowice | **0.83** | **0.92** | **1.13** | **0.97** | **0.97** | **0.45** | **0.70** | **0.67** | **0.51** | 0.36 | **0.78** | **0.58** | **0.74** |
| Kielce | -0.02 | -0.01 | 0.18 | 0.13 | **0.35** | -0.01 | 0.20 | 0.28 | -0.12 | -0.28 | 0.04 | -0.14 | 0.05 |
| Płock | 0.03 | 0.17 | 0.26 | 0.35 | **0.53** | -0.13 | 0.35 | 0.32 | 0.08 | -0.16 | 0.12 | 0.01 | 0.16 |
| Poznań | **0.69** | **0.73** | **0.92** | **1.03** | **0.86** | 0.20 | **0.70** | **0.69** | 0.29 | 0.18 | **0.58** | **0.42** | **0.61** |
| Słubice | 0.17 | 0.33 | 0.43 | **0.66** | **0.63** | 0.13 | **0.45** | **0.47** | 0.07 | -0.05 | 0.17 | 0.01 | **0.29** |
| Tarnów | **1.16** | **1.36** | **1.37** | **1.08** | **1.06** | **0.62** | **0.69** | **0.77** | **0.51** | **0.43** | **0.85** | **0.83** | **0.89** |
| Wrocław | 0.41 | 0.40 | **0.49** | **0.59** | **0.52** | 0.09 | **0.46** | **0.47** | 0.03 | -0.16 | 0.31 | 0.16 | **0.31** |
| **EAST** | | | | | | | | | | | | | |
| Lublin | 0.14 | 0.28 | 0.40 | 0.28 | **0.54** | 0.04 | 0.20 | 0.29 | 0.00 | -0.21 | 0.19 | -0.06 | 0.17 |
| Siedlce | **0.63** | **0.72** | **0.76** | **0.65** | **0.71** | 0.03 | **0.48** | **0.56** | 0.36 | 0.11 | **0.59** | **0.37** | **0.50** |
| Terespol | **0.47** | **0.68** | **0.85** | **0.94** | **0.64** | 0.16 | **0.50** | **0.62** | 0.41 | 0.18 | **0.76** | **0.47** | **0.56** |
| **SOUTH (MOUNTAINS)** | | | | | | | | | | | | | |
| Jelenia Góra | 0.45 | 0.36 | 0.37 | **0.47** | **0.55** | 0.26 | **0.48** | **0.54** | 0.15 | 0.06 | **0.42** | 0.20 | **0.36** |
| Kasprowy W. | 0.38 | 0.43 | 0.21 | 0.22 | 0.42 | 0.17 | 0.30 | **0.62** | -0.02 | -0.36 | 0.28 | 0.30 | **0.24** |
| Kłodzko | 0.25 | 0.19 | 0.08 | 0.40 | **0.36** | 0.17 | 0.30 | **0.43** | -0.05 | **-0.35** | 0.06 | -0.17 | 0.14 |
| Lesko | **0.66** | 0.51 | 0.49 | **0.71** | **0.69** | **0.45** | **0.42** | **0.63** | 0.16 | 0.13 | 0.51 | **0.60** | **0.49** |
| Śnieżka | 0.20 | 0.16 | 0.04 | 0.61 | 0.54 | 0.26 | **0.74** | **1.04** | 0.25 | -0.34 | 0.09 | -0.20 | **0.28** |
| Zakopane | 0.35 | 0.23 | 0.18 | **0.38** | **0.58** | **0.38** | **0.44** | **0.53** | 0.20 | -0.09 | 0.27 | 0.16 | **0.30** |

in **bold** - statistical significance at p≤0.05 level

Table B The values of trends of minimum UTCI per 10 years, 1951-2018

|  | JAN | FEB | MAR | APR | MAY | JUN | JUL | AUG | SEP | OCT | NOV | DEC | YEAR |
| --- | --- | --- | --- | --- | --- | --- | --- | --- | --- | --- | --- | --- | --- |
| **North** | | | | | | | | | | | | | |
| Chojnice | **0.97** | **1.05** | **1.30** | **1.30** | **1.86** | **0.64** | **0.83** | **0.95** | **0.88** | 0.40 | **0.72** | 0.67 | **1.17** |
| Hel | **0.95** | **1.67** | **1.48** | **1.40** | **1.70** | **0.97** | **1.06** | **0.73** | **1.14** | **1.22** | **1.30** | **1.74** | **1.32** |
| Szczecin | 0.66 | **1.47** | **0.89** | **0.75** | **1.12** | 0.41 | **0.56** | **0.74** | **1.07** | **0.64** | **0.75** | **0.91** | **0.97** |
| Świnoujście | **1.04** | 0.84 | 0.33 | 0.44 | **0.94** | 0.59 | 0.38 | **0.79** | **0.69** | 0.16 | 0.52 | **1.11** | 0.56 |
| Ustka | 0.29 | 0.26 | -0.69 | -0.24 | 0.44 | -0.02 | **0.69** | 0.27 | 0.40 | -0.42 | 0.13 | 0.22 | 0.19 |
| **NORTH-EAST** | | | | | | | | | | | | | |
| Białystok | **1.67** | **1.81** | **1.78** | **1.58** | **1.37** | **0.86** | **0.84** | **1.11** | **1.27** | **0.93** | 0.43 | **1.54** | **1.63** |
| Mikołajki | **0.87** | **1.87** | **1.65** | **1.46** | **1.35** | **0.64** | **0.65** | **0.91** | **0.57** | 0.59 | **1.56** | **1.77** | **1.79** |
| Suwałki | **1.50** | **1.86** | **1.52** | **1.50** | **1.42** | 0.63 | 0.55 | **0.77** | **1.03** | **1.22** | **1.77** | **1.65** | **1.85** |
| **CENTER** | | | | | | | | | | | | | |
| Katowice | **1.48** | **1.39** | **2.28** | **1.55** | **1.98** | **1.09** | **1.15** | **0.85** | **1.38** | **0.71** | **1.54** | **1.22** | **1.51** |
| Kielce | 0.41 | 0.55 | **0.99** | 0.40 | **0.86** | 0.42 | 0.07 | 0.05 | 0.06 | 0.02 | 0.50 | **0.67** | **0.55** |
| Płock | 0.32 | **0.73** | **0.94** | 0.68 | **1.23** | 0.26 | **0.74** | **0.70** | **0.57** | 0.10 | **0.93** | **1.03** | **0.71** |
| Poznań | **1.20** | **1.30** | **1.80** | **1.75** | **1.32** | **0.46** | **0.93** | **0.98** | **0.84** | 0.21 | **0.93** | 0.79 | **1.36** |
| Słubice | **1.02** | **1.05** | **1.00** | **1.03** | **1.09** | **0.48** | **0.56** | **0.52** | **0.83** | 0.32 | 0.32 | 0.73 | **1.27** |
| Tarnów | **2.13** | **2.54** | **2.65** | **1.83** | **2.07** | **1.29** | **1.08** | **0.95** | **1.38** | **1.10** | **1.88** | **2.00** | **2.19** |
| Wrocław | **1.29** | **0.99** | **1.37** | 0.70 | **1.53** | **0.70** | **0.93** | **0.93** | **0.85** | 0.56 | **0.77** | 0.59 | **1.26** |
| **EAST** | | | | | | | | | | | | | |
| Lublin | 0.34 | **1.09** | 0.54 | 0.38 | 0.42 | 0.40 | 0.15 | 0.17 | 0.45 | -0.14 | 0.48 | 0.42 | **0.79** |
| Siedlce | **0.89** | **1.50** | **1.58** | **1.16** | **1.55** | **0.84** | **0.64** | **0.65** | **0.99** | 0.38 | **0.91** | **0.97** | **1.45** |
| Terespol | **1.06** | **1.41** | **1.14** | **1.55** | **1.26** | **1.09** | 0.41 | **0.98** | **0.88** | 0.54 | **1.13** | **1.55** | **1.36** |
| **SOUTH (MOUNTAINS)** | | | | | | | | | | | | | |
| Jelenia Góra | **1.46** | **1.40** | **1.57** | **0.87** | **1.41** | **0.64** | **0.92** | **0.88** | **1.28** | **0.75** | 0.67 | **1.23** | **1.58** |
| Kasprowy W. | **0.96** | **0.75** | **0.89** | 0.58 | **1.36** | 0.70 | 0.51 | **1.21** | 0.69 | -0.41 | 0.82 | 0.36 | **0.79** |
| Kłodzko | -0.23 | 0.63 | 0.37 | 0.18 | **1.06** | **0.76** | **0.53** | **0.61** | **0.59** | -0.24 | 0.07 | 0.08 | 0.07 |
| Lesko | **2.06** | **1.11** | **1.32** | **1.03** | **1.30** | **1.35** | 0.60 | **1.38** | **0.77** | 0.33 | **1.37** | **1.34** | **1.62** |
| Śnieżka | 0.41 | 0.44 | **0.56** | 0.13 | 0.21 | -0.15 | **0.90** | **1.21** | **0.88** | -0.46 | -0.23 | 0.06 | 0.27 |
| Zakopane | **1.22** | **0.99** | **1.04** | **0.66** | **1.02** | **0.95** | **0.73** | **0.64** | **1.09** | 0.56 | **0.98** | **1.22** | **1.47** |

in **bold** - statistical significance at p≤0.05 level

Table C. The values of trends of maximum UTCI per 10 years, 1951-2018

|  | JAN | FEB | MAR | APR | MAY | JUN | JUL | AUG | SEP | OCT | NOV | DEC | YEAR |
| --- | --- | --- | --- | --- | --- | --- | --- | --- | --- | --- | --- | --- | --- |
| **North** | | | | | | | | | | | | | |
| Chojnice | -0.28 | -0.47 | -0.16 | 0.19 | 0.01 | -0.01 | 0.09 | 0.07 | -0.27 | -0.35 | -0.08 | **-0.60** | 0.05 |
| Hel | -0.01 | -0.26 | 0.09 | 0.09 | 0.23 | -0.31 | -0.08 | 0.00 | **-0.41** | -0.26 | -0.17 | -0.10 | -0.05 |
| Szczecin | 0.16 | 0.12 | 0.24 | 0.23 | 0.29 | 0.17 | 0.30 | **0.43** | -0.32 | 0.11 | 0.35 | 0.01 | **0.32** |
| Świnoujście | **0.78** | 0.45 | **0.82** | **0.63** | **0.74** | 0.13 | **0.50** | **0.44** | 0.01 | 0.12 | **0.51** | 0.34 | **0.45** |
| Ustka | 0.12 | -0.17 | -0.17 | 0.22 | **0.60** | -0.19 | 0.02 | 0.16 | -0.23 | -0.46 | -0.16 | **-0.47** | 0.16 |
| **NORTH-EAST** | | | | | | | | | | | | | |
| Białystok | **0.48** | 0.21 | 0.23 | 0.35 | 0.24 | 0.13 | 0.21 | **0.36** | 0.13 | 0.00 | 0.43 | 0.11 | **0.28** |
| Mikołajki | 0.21 | 0.18 | 0.02 | 0.50 | 0.28 | **0.36** | 0.28 | **0.41** | 0.05 | 0.18 | 0.13 | -0.38 | **0.34** |
| Suwałki | **0.54** | 0.12 | 0.53 | **0.92** | **0.57** | **0.57** | **0.56** | **0.64** | 0.12 | **0.54** | 0.42 | 0.12 | **0.53** |
| **CENTER** | | | | | | | | | | | | | |
| Katowice | 0.27 | 0.47 | 0.37 | **0.45** | 0.13 | 0.29 | **0.35** | **0.30** | -0.06 | 0.17 | 0.26 | -0.05 | **0.35** |
| Kielce | 0.09 | -0.02 | 0.02 | 0.34 | 0.09 | 0.13 | 0.15 | 0.26 | -0.27 | -0.05 | 0.05 | **-0.50** | 0.19 |
| Płock | **-0.58** | -0.31 | -0.12 | -0.03 | -0.01 | -0.03 | -0.03 | 0.21 | **-0.46** | -0.39 | 0.02 | **-0.65** | 0.16 |
| Poznań | 0.30 | 0.12 | 0.31 | 0.43 | 0.34 | 0.35 | 0.29 | 0.07 | -0.20 | **-0.49** | 0.45 | -0.14 | 0.18 |
| Słubice | -0.15 | -0.12 | 0.08 | 0.10 | **0.48** | 0.24 | 0.28 | 0.28 | -0.40 | -0.32 | -0.03 | -0.37 | 0.25 |
| Tarnów | 0.49 | **0.67** | 0.38 | **0.52** | **0.60** | **0.35** | **0.41** | **0.46** | -0.09 | 0.19 | 0.19 | 0.03 | **0.39** |
| Wrocław | 0.13 | 0.03 | 0.03 | 0.28 | 0.22 | 0.29 | 0.24 | 0.24 | -0.34 | -0.15 | -0.02 | -0.53 | **0.27** |
| **EAST** | | | | | | | | | | | | | |
| Lublin | -0.01 | 0.16 | 0.19 | 0.35 | 0.38 | 0.06 | 0.18 | 0.30 | -0.11 | -0.01 | 0.14 | -0.40 | 0.16 |
| Siedlce | 0.37 | 0.01 | 0.26 | 0.15 | 0.22 | -0.07 | 0.29 | **0.36** | -0.12 | 0.01 | 0.20 | -0.12 | **0.22** |
| Terespol | 0.05 | 0.27 | 0.51 | 0.27 | 0.25 | 0.14 | 0.27 | **0.42** | 0.12 | -0.17 | 0.29 | -0.03 | **0.30** |
| **SOUTH (MOUNTAINS)** | | | | | | | | | | | | | |
| Jelenia Góra | 0.22 | -0.43 | 0.13 | 0.11 | 0.20 | 0.23 | 0.24 | **0.35** | -0.38 | -0.02 | 0.01 | -0.46 | 0.24 |
| Kasprowy W. | **-0.70** | -0.71 | -0.09 | -0.48 | -0.24 | -0.05 | -0.06 | -0.07 | -0.35 | -0.17 | -0.34 | -0.45 | -0.09 |
| Kłodzko | -0.12 | -0.09 | 0.05 | 0.23 | -0.05 | 0.17 | 0.16 | -0.04 | -0.39 | -0.26 | -0.27 | -0.32 | 0.04 |
| Lesko | 0.16 | -0.02 | -0.02 | 0.08 | 0.31 | 0.18 | 0.20 | 0.24 | 0.02 | -0.17 | 0.05 | **-0.52** | 0.21 |
| Śnieżka | -0.64 | -0.43 | -0.71 | 0.50 | 0.32 | 0.44 | 0.33 | -0.02 | -0.11 | -0.60 | -0.61 | **-1.05** | 0.20 |
| Zakopane | 0.37 | 0.07 | -0.14 | 0.20 | **0.50** | **0.50** | **0.32** | **0.34** | -0.16 | 0.00 | 0.07 | 0.06 | 0.35 |

in **bold** - statistical significance at p≤0.05 level

Table D. The values of trends of cold stress days, with UTCI ≤-13.0°C per 10 years, 1951-2018

|  | JAN | FEB | MAR | APR | MAY | JUN | JUL | AUG | SEP | OCT | NOV | DEC | YEAR |
| --- | --- | --- | --- | --- | --- | --- | --- | --- | --- | --- | --- | --- | --- |
| **North** | | | | | | | | | | | | | |
| Chojnice | -0.07 | -0.50 | **-0.70** | **-0.41** | **-0.08** |  |  |  | 0.00 | -0.07 | -0.41 | -0.24 | **-2.47** |
| Hel | **-1.28** | **-1.21** | **-0.91** | **-0.39** | **-0.07** |  |  |  |  | -0.11 | **-0.82** | **-1.44** | **-6.22** |
| Szczecin | **-0.85** | **-0.76** | **-0.59** | -0.09 | -0.01 |  |  |  | -0.01 | -0.04 | **-0.33** | **-0.54** | **-3.23** |
| Świnoujście | **-1.20** | **-0.85** | -0.37 | -0.17 | 0.00 | 0.00 | 0.00 |  |  | 0.03 | -0.23 | **-0.71** | **-3.50** |
| Ustka | 0.09 | -0.14 | 0.23 | 0.05 | 0.05 |  |  |  | 0.00 | 0.09 | -0.24 | -0.14 | -0.01 |
| **NORTH-EAST** | | | | | | | | | | | | | |
| Białystok | **-1.22** | **-1.10** | **-0.91** | **-0.24** | -0.01 | -0.01 |  |  | -0.02 | **-0.16** | **-1.07** | **-1.01** | **-5.75** |
| Mikołajki | **-0.81** | **-0.99** | **-0.86** | **-0.26** | -0.01 |  |  |  | -0.01 | -0.12 | **-0.68** | **-0.70** | **-4.44** |
| Suwałki | **-0.87** | **-1.13** | **-1.38** | **-0.54** | **-0.09** | 0.00 |  |  | -0.03 | **-0.25** | **-1.39** | **-1.25** | **-6.94** |
| **CENTER** | | | | | | | | | | | | | |
| Katowice | **-1.15** | **-1.21** | **-1.20** | **-0.20** | -0.03 |  |  |  |  | **-0.07** | **-0.54** | **-1.06** | **-5.45** |
| Kielce | 0.14 | 0.03 | -0.38 | 0.01 | 0.00 |  |  |  |  | -0.03 | 0.00 | 0.01 | -0.22 |
| Płock | -0.33 | -0.51 | **-0.54** | -0.09 | -0.02 |  |  |  |  | 0.00 | -0.33 | -0.20 | **-2.03** |
| Poznań | **-1.20** | **-1.15** | **-1.10** | **-0.40** | -0.04 |  |  |  |  | -0.01 | **-0.53** | **-1.04** | **-5.47** |
| Słubice | -0.34 | **-0.49** | **-0.35** | **-0.09** | -0.01 |  |  |  |  | -0.01 | -0.15 | -0.24 | **-1.69** |
| Tarnów | **-1.42** | **-1.33** | **-1.01** | **-0.10** | -0.01 |  |  |  |  | **-0.06** | **-0.54** | **-1.08** | **-5.55** |
| Wrocław | -0.52 | **-0.64** | **-0.64** | -0.08 | **-0.02** |  |  |  | -0.01 | -0.04 | -0.28 | **-0.52** | **-2.76** |
| **EAST** | | | | | | | | | | | | | |
| Lublin | -0.25 | -0.22 | -0.30 | -0.02 | -0.01 |  |  |  | -0.01 | -0.04 | -0.21 | 0.09 | -0.97 |
| Siedlce | **-0.96** | **-0.94** | **-0.86** | -0.13 | -0.01 | -0.01 |  |  |  | -0.04 | **-0.62** | **-0.82** | **-4.38** |
| Terespol | **-0.83** | **-0.95** | **-0.77** | **-0.25** | -0.03 |  |  |  | -0.01 | -0.06 | **-0.64** | **-0.94** | **-4.47** |
| **SOUTH (MOUNTAINS)** | | | | | | | | | | | | | |
| Jelenia Góra | **-0.56** | **-0.62** | **-0.42** | -0.14 | -0.03 |  |  |  | **-0.02** | -0.05 | **-0.34** | -0.25 | **-2.43** |
| Kasprowy W. | 0.14 | 0.10 | 0.07 | -0.37 | **-0.51** | -0.26 | -0.26 | **-0.29** | -0.13 | 0.01 | -0.10 | 0.22 | -1.39 |
| Kłodzko | -0.44 | -0.35 | -0.19 | -0.04 |  |  |  |  |  | 0.02 | -0.14 | 0.00 | -1.14 |
| Lesko | **-0.76** | **-0.66** | **-0.66** | -0.09 | -0.02 | -0.01 |  |  | -0.01 | -0.08 | **-0.61** | **-1.07** | **-3.49** |
| Śnieżka | 0.06 | 0.08 | 0.11 | -0.51 | -0.50 | -0.05 | -0.42 | **-0.75** | -0.26 | 0.21 | 0.22 | 0.16 | -1.65 |
| Zakopane | **-0.42** | **-0.42** | **-0.37** | -0.04 | -0.01 |  |  |  |  | -0.02 | -0.19 | **-0.32** | **-1.78** |

in **bold** - statistical significance at p≤0.05 level

Table E. The values of trends of heat stress days, with UTCI >32.0°C per 10 years, 1951-2018

|  | APR | MAY | JUN | JUL | AUG | SEP | YEAR |
| --- | --- | --- | --- | --- | --- | --- | --- |
| **North** | | | | | | | |
| Chojnice | 0.01 | **-0.02** | 0.02 | 0.08 | -0.06 | -0.06 | -0.03 |
| Hel |  |  | 0.01 | 0.01 | -0.06 | -0.01 | -0.05 |
| Szczecin | -0.01 | 0.00 | 0.05 | 0.20 | **0.27** | 0.00 | 0.51 |
| Świnoujście |  | 0.02 | 0.03 | **0.22** | **0.18** | -0.01 | **0.44** |
| Ustka | 0.00 | -0.01 | -0.01 | 0.04 | 0.00 | -0.02 | 0.01 |
| **NORTH-EAST** | | | | | | | |
| Białystok | -0.01 | 0.02 | 0.02 | 0.30 | 0.23 | -0.01 | **0.56** |
| Mikołajki | 0.01 | 0.03 | 0.07 | 0.21 | **0.35** | 0.00 | 0.67 |
| Suwałki | 0.01 | 0.01 | **0.12** | **0.39** | **0.27** | -0.01 | **0.80** |
| **CENTER** | | | | | | | |
| Katowice |  | 0.03 | 0.14 | **0.35** | **0.37** | -0.03 | **0.86** |
| Kielce |  | 0.00 | 0.01 | 0.10 | 0.16 | -0.07 | 0.20 |
| Płock | -0.01 | -0.01 | -0.12 | 0.00 | 0.07 | -0.01 | -0.07 |
| Poznań | -0.01 | 0.02 | **0.19** | 0.32 | 0.15 | -0.03 | **0.65** |
| Słubice | -0.01 | 0.04 | 0.14 | 0.19 | 0.26 | -0.08 | 0.54 |
| Tarnów | 0.02 | **0.13** | **0.37** | **0.69** | **0.73** | -0.01 | **1.93** |
| Wrocław | -0.01 | 0.04 | 0.13 | 0.20 | 0.24 | -0.03 | 0.58 |
| **EAST** | | | | | | | |
| Lublin | -0.01 | 0.03 | 0.05 | **0.34** | 0.24 | -0.03 | **0.63** |
| Siedlce |  | 0.04 | -0.09 | 0.22 | 0.25 | -0.04 | 0.38 |
| Terespol | 0.01 | 0.05 | 0.06 | **0.44** | **0.51** | **0.06** | **1.14** |
| **SOUTH (MOUNTAINS)** | | | | | | | |
| Jelenia Góra | -0.01 | 0.00 | 0.12 | 0.13 | 0.23 | **-0.08** | 0.38 |
| Kasprowy W. |  |  |  |  |  |  |  |
| Kłodzko | -0.01 | 0.01 | 0.09 | 0.12 | 0.13 | -0.03 | 0.32 |
| Lesko |  | 0.03 | 0.03 | 0.20 | **0.38** | -0.01 | **0.62** |
| Śnieżka |  |  |  |  |  |  |  |
| Zakopane |  |  | **0.07** | 0.09 | 0.13 | 0.01 | **0.30** |

in **bold** - statistical significance at p≤0.05 level
